# The influence of cultural and religious factors on cross-national variations in the prevalence of chronic back and neck pain: an analysis of data from the global burden of disease 2019 study

**DOI:** 10.3389/fpain.2023.1189432

**Published:** 2023-05-25

**Authors:** Ravi Philip Rajkumar

**Affiliations:** Department of Psychiatry, Jawaharlal Institute of Postgraduate Medical Education and Research, Pondicherry, India

**Keywords:** chronic pain, low back pain, neck pain, religion, culture, spirituality

## Abstract

**Introduction:**

Low back pain and neck pain are among the most commonly reported forms of chronic pain worldwide, and are associated with significant distress, disability and impairment in quality of life. Though these categories of pain can be analyzed and treated from a biomedical perspective, there is evidence that they are both related to psychological variables such as depression and anxiety. The experience of pain can be significantly influenced by cultural values. For example, cultural beliefs and attitudes can influence the meaning attached to the experience of pain, the responses of others to a sufferer's pain, and the likelihood of seeking medical care for particular symptoms. Likewise, religious beliefs and practices can influence the both experience of pain and the responses to it. These factors have also been associated with variations in the severity of depression and anxiety.

**Methods:**

In the current study, data on the estimated national prevalence of both low back pain and neck pain, obtained from the 2019 Global Burden of Disease Study (GBD 2019), is analyzed in relation to cross-national variations in cultural values, as measured using Hofstede's model (*n* =115 countries) and in religious belief and practice, based on the most recent Pew Research Center survey (*n* = 105 countries). To address possible confounding factors, these analyses were adjusted for variables known to be associated with chronic low back or neck pain, namely smoking, alcohol use, obesity, anxiety, depression and insufficient physical activity.

**Results:**

It was found that the cultural dimensions of Power Distance and Collectivism were inversely correlated with the prevalence of chronic low back pain, and Uncertainty Avoidance was inversely correlated with the prevalence of chronic neck pain, even after adjustment for potential confounders. Measures of religious affiliation and practice were negatively correlated with the prevalence of both conditions, but these associations were not significant after adjusting for cultural values and confounders.

**Discussion:**

These results highlight the existence of meaningful cross-cultural variations in the occurrence of common forms of chronic musculoskeletal pain. Psychological and social factors that could account for these variations are reviewed, along with their implications for the holistic management of patients with these disorders.

## Introduction

Pain is a universal defensive mechanism present in both animals and humans. From an evolutionary perspective, the mechanisms involved in the perception of pain and the response to it have been conserved due to the survival advantages they confer. These advantages include withdrawal from injurious or noxious stimuli, the promotion of wound healing, and the ability to signal danger or a need for help (1). Molecular mechanisms involved in the perception of such stimuli, and withdrawal from them, have been documented even in invertebrate organisms, such as mollusks (2). However, in many cases, humans experience pain that is persistent, severe, disabling, and not seemingly related to any acute risk of tissue damage or injury. This condition is referred to as chronic pain, and it is one of the leading causes of disability around the world (3). One of the commonest types of chronic pain occurs in relation to the components of the musculoskeletal system, and is referred to as chronic musculoskeletal pain (4). For example, meta-analysis of 122 publications from low- and middle-income countries found that the prevalence of chronic pain was 35% in the general population and 56% in elderly adults. Among the population of those diagnosed with a chronic pain, musculoskeletal pain was the commonest diagnosis, accounting for over 40% of all cases in these countries (5). Similar results were obtained in a large cross-national study of older adults from Europe, in which 36% of respondents suffered from chronic musculoskeletal pain (6). Chronic musculoskeletal pain is a particularly common problem among adults in active employment, affecting over 60%–70% of this population, and frequently leading to reduced work performance, loss of income, or unemployment (7–9).

The category of chronic musculoskeletal pain is itself a broad one, including such entities as chronic widespread pain, shoulder pain, low back pain and neck pain. However, low back pain and neck pain are among the commonest types within this category. The prevalence of chronic low back pain has been estimated at around 11% in the general population and 20%–36% in older adults (10, 11), while the lifetime prevalence of chronic neck pain has been estimated at around 48% (12). Moreover, these two types of pain frequently co-occur: it has been found that around 30%–55% of patients with chronic low back pain also experience neck pain (13). Chronic back and neck pain also show evidence of familial aggregation, which suggests that they may share genetic risk factors (14).

The cause of most cases of chronic low back or neck pain is largely unknown. Many hypotheses have been advanced to explain the pathogenesis of these conditions, including mechanical or degenerative changes in local musculoskeletal structures, increased inflammatory activity, increased sensitization to pain at the level of the central nervous system and impairments in sensorimotor control (15–18). Despite much active research in this area, the evidence supporting these hypotheses is often inconsistent, and there is a paucity of evidence to guide the choice of safe and effective treatments in these patients (18–20). Notwithstanding this knowledge gap, most patients with these disorders are treated with pharmacological therapies, such as analgesics and antidepressants (21), and some undergo surgical procedures with the aim of correcting problems of a mechanical or degenerative nature (22). However, these treatment modalities are often of limited efficacy (22–24), and some of them, such as opioid analgesics and surgery, are associated with a significant risk of harm (25, 26).

Owing to these limitations, there is also a significant amount of interest in the role of psychological and social factors in the onset, persistence, and outcome of chronic low back and neck pain. From a psychological perspective, both these conditions appear to be genetically linked to depression (14). Depressive disorders are more common in patients with these disorders than in the general population (27, 28), and depression has been found to predict functional outcomes in these patients (29, 30). Apart from depression, other negative emotional states such as anxiety and anger have also been associated with both the occurrence of these conditions, and the level of disability associated with them (31–33). Psychological stress, particularly when chronic in nature, has been associated with both these types of pain (33, 34), and there is some evidence that patients with these conditions are more sensitive to the effects of stress (35, 36). The cognitive styles of individuals with these pain disorders also appear to differ from those of healthy controls in certain key aspects, such as reduced flexibility (37) and exaggerated ideas or beliefs regarding the causes or likely consequences of their pain (38). In the light of these findings, a wide range of psychologically-oriented therapies, based on cognitive- or mindfulness-related principles, have been tried in patients with back or neck pain, and have been found to reduce both subjective perceptions of pain severity and quality of life (39, 40).

These psychological findings should, like the biological models discussed earlier, not be viewed in isolation, but as part of a biopsychosocial approach to the pathogenesis and management of chronic musculoskeletal pain (41). Cultural factors can influence individuals' mental health and psychological responses to pain, as well as community- and workplace-related factors that can either facilitate or hinder recovery from chronic back and neck pain. For example, cultural variations in individualism-collectivism—that is, in the extent to which the society accords importance to the individual or to the larger community—have been associated with regional or cross-national variations in the prevalence of depression (42), in emotional responses to a given experiences (43), in coping with stress or adversity (44), and in the manner in which others in the patient's environment respond to their pain (45). Cross-cultural variations in cognitive flexibility, which is significantly associated with chronic pain, have also been shown to exist from childhood onwards (46). Similarly, cultural differences in power distance, which measures the level of hierarchy and the tolerance of inequality in a given society, are associated with cross-national variations in workplace culture and stress (47, 48), which are risk factors for chronic neck pain (49). Culture can also influence how individuals experience and report chronic pain and the disability associated with it, requiring adaptations in the instruments used to measure these variables (50). Moreover, culture can also influence the type of medical care received by these patients. For example, a study of prescribing trends in Europe found that cultural dimensions, such as individualism-collectivism, long-term orientation, and indulgence vs. restraint, influenced variations in the prescriptions of drugs used to manage these types of pain, such as duloxetine and pregabalin, between countries (51). In a meta-analytic review of studies of chronic pain, it was observed that three cultural dimensions—power distance, individualism-collectivism, and indulgence vs. restraint—mediated the association between fear-related avoidance and the severity of pain (52).

Religion and spirituality, as integral parts of culture that shape most aspects of human existence, have also been associated with certain aspects of these types of pain. From a theoretical perspective, religious or spiritual coping has been postulated to exert a beneficial effect both on pain and on the negative mood states, such as depression, that are both caused by and exacerbate it (53). However, due to the relatively small number of studies examining the association between religiosity and musculoskeletal pain, results have often been inconsistent or even conflicting in real-world settings. A study of elderly adults with chronic low back pain found that self-reported religious coping was negatively associated with the intensity of pain and positively associated with pain acceptance, suggesting a protective effect (54). A subsequent systematic review confirmed the association between religious beliefs or attitudes and pain acceptance, but also reported possible negative outcomes such as worse pain-related cognitions and mood states; however, this review acknowledged that most of the available evidence was of low quality and possibly biased (55).

One of the major reasons for variations across studies is that “religion” and “spirituality” are not unitary constructs: different religions, or even different sub-groups or sects within a religion, differ substantially in the significance that they attach to pain, the responses to suffering that are considered appropriate, and the extent of support provided to an individual suffering from chronic pain. Moreover, cultural beliefs and attitudes that are not directly related to religion can act as confounding factors. For example, a study of Ghanaian adults revealed that their religion was a source of hope and support in the face of chronic back pain; on the other hand, many of these adults had culturally-derived maladaptive beliefs related to pain, which were not specifically related to their religion (56). Similar results regarding the positive role of religion were reported in a study of Spanish and Brazilian patients with back pain (57). A study of Arab Muslim patients found that religion was associated with both active and passive coping strategies, with the former having a more positive effect on well-being (58). In a study of office workers with chronic low back pain from Thailand, respondents' self-reported level of adherence to Buddhist beliefs and practices was associated with lower levels of depression and lower salivary cortisol—a marker of stress—but not with any significant reduction in disability (59). Two studies from Nigeria further underline the complexity of the associations between various aspects of religion and these types of pain. In the first, it was found that low back pain was more common in Christian than in Muslim adolescents (60). In the second, it was observed that “unconventional” health practitioners, who are often the first point of contact for Nigerian patients with chronic low back pain, differ significantly in the nature of the guidance they offer: practitioners of herbal medicine seemed to endorse passive coping and adherence to pharmacotherapy (both herbal and allopathic), while pastors favoured spiritual explanations of the cause of back pain, and accordingly offered spiritual healing to their clients, but also encouraged pain acceptance and fostered resilience in their clients (61). Finally, it should be noted that these relationships are not unidirectional: the presence of chronic low back or neck pain can interfere with body posture and mobility, leading to difficulties in adhering to certain religious practices. This can in turn lead to psychological distress, which might exacerbate the underlying pain (62, 63).

The foregoing discussion makes it clear that the management of chronic neck and back pain, particularly in non-“Western” settings, requires a careful integration of both cultural factors and of religion/spirituality into treatment approaches (41, 53). Such a wish is often reported by patients themselves (64). To achieve this effectively, it would be useful to identify those cultural factors, or those aspects of religious belief or practice, that are meaningfully associated with variations in the occurrence of these disorders. Such an analysis would gain additional validity if an attempt was made to correct for lifestyle, medical and psychosocial factors, such as obesity, physical activity and depression, that are themselves associated with chronic low back and neck pain (27, 49).

The aim of the current study is to examine whether cross-national variations in cultural values, and in self-reported religious affiliation and practice, are associated with significant variations in the prevalence of chronic low back pain and neck pain, as estimated by the 2019 Global Burden of Disease Study. To minimize the risk of spurious correlations, this study will also attempt to correct for the effects of factors independently associated with these disorders, namely tobacco use, alcohol use, depression, anxiety, obesity and insufficient physical activity.

## Materials and methods

The current study was a cross-sectional, cross-national, ecological association study. The outcome variables of interest were the estimated prevalence of chronic low back pain and neck pain, obtained from the 2019 Global Burden of Disease Study (GBD 2019). The independent variables of interest were: (a) scores measuring specific cultural values at a national level, namely the Global Collectivism Index (GCI) and Hofstede's six cultural dimensions, and (b) self-reported levels of religious affiliation, belief and practice, based on the most recent Pew Research Center report. The confounding/interacting variables studies were the estimated prevalence of depression, anxiety and obesity; the percentage of the population of each country reporting tobacco or alcohol use; and the estimated proportion of adults in each country whose level of physical activity was considered insufficient.

### Data sources

The Global Burden of Disease studies provide cross-national estimates of the incidence, prevalence and disability associated with a wide range of diseases and disorders, including musculoskeletal disorders, for 204 countries and territories (65). In this group of disorders, separate estimates have been made for the distribution of both chronic low back pain and chronic neck pain in each country. These estimates are available through database queries from the Global Burden of Disease Collaborative Network, which is hosted by the Institute for Health Metrics and Evaluation (IHME) located in Seattle (66). To minimize the confounding effect of variations in population demographics, such as higher life expectancies leading to higher prevalence estimates, age-standardized estimates of prevalence were obtained for both disorders and used in this study.

Two measures of cross-national variations in culture were used in this study. The first, the Global Collectivism Index (CGI), is a measure of individualism-collectivism that has been computed for 188 countries and territories with the explicit aim of providing a measure of this value that is valid regardless of a country's income grouping or geographical location. The GCI is a composite index calculated based on five factors: fertility rate, household size, marriage-to-divorce ratio, religiosity, collective transportation, and attitudes favoring interdependence. Higher scores on the GCI indicate a collectivistic cultural orientation, while lower values indicate an individualistic orientation. GCI scores range from a maximum of 1.92 (Somalia) to a minimum of −1.85 (Monaco) and were retrieved from the original publication describing the development of this index (67). The second measure of a nation's culture was the six-factor model developed by Geert Hofstede and his colleagues. This model describes each nation's culture in terms of ordinal scores, rated from 0 to 100, on six roughly orthogonal dimensions: Power Distance, Individualism-Collectivism, Masculinity-Femininity, Uncertainty Avoidance, Long-Term Orientation and Indulgence-Restraint. A full definition of each of these dimensions is provided in Table 1 below. Hofstede's model was used for this study because (a) it captures a wide range of cultural values beyond individualism-collectivism, (b) data on this model is available for a large number of countries (*n *= 116), and (c) prior research has established a tentative connection between three of Hofstede's dimensions and specific aspects of chronic pain (52). The Hofstede dimension scores are based on survey data from individuals across various countries, and are available through database queries from the Hofstede Insights database (68).

**Table 1:** Study variables, definition, sources and availability.

Variable	Definition	Data source	Availability
Prevalence of chronic low back pain (CLBP)	Age-standardized prevalence of chronic low back pain (%)	Global Burden of Disease 2019 study	204 countries
Prevalence of chronic neck pain (CNP)	Age-standardized prevalence of chronic neck pain (%)	Global Burden of Disease 2019 study	204 countries
Global Collectivism Index (GCI)	A continuous measure of national individualism-collectivism. Higher scores indicate higher collectivism	Original publication	185 countries
Hofstede index of Power Distance (HOF-PD)	A continuous measure of the extent to which inequalities in the distribution of power are expected and accepted in a society. Higher scores indicate a higher power distance.	Hofstede Insights database	116 countries
Hofstede Index of Individualism-Collectivism (HOF-IC)	A continuous measure of the extent to which a society privileges independence or interdependence. Higher scores indicate higher individualism (i.e, scoring order is the reverse of the GCI).	Hofstede Insights database	116 countries
Hofstede Index of Masculinity-Femininity (HOF-MF)	A continuous measure of the extent to which social values favour competition in contrast to cooperation. Higher scores indicate higher masculinity.	Hofstede Insights database	116 countries
Hofstede Index of Uncertainty Avoidance (HOF-UAI)	A continuous measure of the extent to which a society tolerates uncertain situations and uses beliefs or social institutions to handle them. Higher scores indicate higher avoidance of uncertainty.	Hofstede Insights database	116 countries
Hofstede Index of Long-Term Orientation (HOF-LTO)	A continuous measure of a society's preference for tradition as opposed to modernity. Higher scores indicate a more “future” (long-term) orientation.	Hofstede Insights database	100 countries
Hofstede Index of Indulgence vs. Restraint (HOF-IVR)	A continuous measure of a society's willingness to allow individuals to engage in pleasurable activities, as opposed to restraining their desires and impulses. Higher scores indicate greater indulgence	Hofstede Insights database	96 countries
Religious affiliation (REL-AFF)	Percentage of respondents reporting affiliation to a particular religion	Pew Research Center report	105 countries
Religion—Weekly Attendance (REL-ATT)	Percentage of respondents reporting at least weekly attendance of religious services or rituals	Pew Research Center report	101 countries
Religion—Daily Prayer (REL-PRAY)	Percentage of respondents reporting the practice of daily prayer	Pew Research Center	104 countries
Religion—Importance (REL-IMP)	Percentage of respondents reporting that religion is “important” or “very important” to them	Pew Research Center	105 countries
Prevalence of depression (DEP)	Age-standardized prevalence of depressive disorders (%)	Global Burden of Disease 2019 study	204 countries
Prevalence of anxiety disorders (ANX)	Age-standardized prevalence of anxiety disorders (%)	Global Burden of Disease 2019 study	204 countries
Prevalence of tobacco use (TOB)	Percentage of adults estimated to use tobacco	Global Health Observatory	163 countries
Prevalence of alcohol use (ALC)	Percentage of adults estimated to consume alcohol	Global Health Observatory	188 countries
Prevalence of obesity (OB)	Percentage of adults estimated to have a body mass index (BMI) greater than 30 kg/m^2^	Global Health Observatory	191 countries
Prevalence of insufficient physical activity (IN-PA)	Percentage of adults whose level of physical activity is estimated to be inadequate	Global Health Observatory	162 countries

All study variables refer to country-level estimates.

Data on religion was obtained from the most recent (2018) Pew Research Center report, entitled “The age gap in religion around the world” (69). This report, based on data from multiple surveys conducted in the period 2008–2017, includes data from 105 countries. For each country, the following data is available: (a) percentage of respondents reporting any religious affiliation, (b) percentage reporting daily prayer, (c) percentage reporting weekly attendance at religious services or rituals, and (d) percentage reporting that they consider religion important in their lives. These four parameters were used as measures of religious belief and practice in the current study.

Though several lifestyle and psychosocial variables have been associated with chronic back and neck pain, reliable cross-national data is available for only some of them. Therefore, possible confounding factors were selected for inclusion in the current study based on two criteria: (a) clear evidence of an association between the variable in question and either low back pain or neck pain, based either on systematic reviews or large observational studies of good quality, and (b) availability of reliable data on the variable for at least 100 countries. Based on these criteria, the following variables were included in the analyses of the current study: estimated prevalence of depression, anxiety disorders and obesity (all age-standardized), estimated percentage of adults with insufficient physical activity, and estimated percentage of the population using alcohol and tobacco. Data on these variables was obtained from the World Health Organization's Global Health Observatory (70).

A complete list of all the study variables, their definition, their sources and availability is provided in Table 1.

### Data analysis

In the initial step of the data analysis, direct bivariate correlations (Pearson's *r*) were used to examine the strength and direction of the associations between cultural and religious variables and the estimated prevalence of chronic low back pain and neck pain. For these analyses, Bonferroni's correction was applied to minimize the risk of false-positive results. The correlations between both these sets of variables and the potential confounding factors included in this study—namely depression, anxiety, obesity, insufficient physical activity, and alcohol and tobacco use—were also examined using the same method.

In the second step, partial bivariate correlations (Pearson's partial *r*) were used to examine whether any identified associations between cultural and religious factors and the prevalence of back or low neck pain remained significant after adjusting for potential confounders. Confounders were selected in these analyses if they were significantly correlated with either set of variables in the previous step. Both direct and partial correlation analyses were two-tailed, and a *p* value of < .05 (with Bonferroni's correction for the direct bivariate correlations) was considered significant.

When reporting bivariate and partial correlations, the strength of each association was reporting according to standard guidelines for psychosocial research (71) as follows: absolute value of *r* (|*r*|) < 0.1, zero (no) correlation; |*r*| = 0.1 to 0.39, weak correlation; |*r*| = 0.4 to 0.69, moderate correlation; |*r*| ≥ 0.7, strong correlation.

In the third step, multivariate linear regression analyses were carried out to identify the consistency and strength of the associations between cultural and religious factors and the prevalence of both types of pain. All variables that were significantly associated with either type of pain at *p *< .05 or less in the bivariate analyses were included in the regression analyses. To address the issue of possible multicollinearity between variables, variance inflation factors (VIFs) were computed for all independent variables. If the VIF exceeded 4 for any of these variables, it was excluded and the analysis repeated until the VIF for all independent variables was ≤ 4.

## Results

Data on the estimated prevalence of chronic low back and neck pain was available for 204 countries and territories. The estimated prevalence of low back pain was 8.11 ± 1.61%, with a maximum of 13.47% (United States) and a minimum of 5.37% (India). The estimated prevalence of neck pain was 2.28 ± 1.14%, with a maximum of 5.55% (Philippines) and a minimum of 0.96% (New Zealand). There was a moderate positive correlation between the prevalence of these types of pain (*r *= .55, *p *< .001). Descriptive statistics for the other study variables are presented in Supplementary Material Table S1.

### Bivariate correlations between culture, religion, and the prevalence of low back and neck pain

Unadjusted bivariate correlations between cultural variables and the prevalence of chronic low back and neck pain are presented in Table 2. In these analyses, the prevalence of chronic low back pain was negatively correlated with the GCI and positively correlated with Hofstede's index of individualism-collectivism; in other words, the prevalence of chronic low back pain was negatively associated with collectivistic cultural values, even after applying Bonferroni's correction. The strength of this association was moderate. Low back pain was also negatively correlated with Hofstede's index of Power Distance, and positively correlated with Uncertainty Avoidance and Long-Term Orientation. However, of these three, only the associations with Power Distance and Long-Term Orientation survived correction for multiple comparisons.

**Table 2:** Bivariate correlations between dimensional measures of cultural values and the national prevalence of chronic low back and neck pain.

Variable	1 CLBP	2 CNP	3 GCI	4 HOF-PD	5 HOF-IC	6 HOF-MF	7 HOF-UAI	8 HOF-LTO	9 HOF-IVR
**1**	–	.55 (<.001)**	-.64 (<.001)**	-.32 (<.001)**	.55 (<.001)**	.07 (.433)	.21 (.026)*	.45 (<.001)**	-.09 (.393)
**2**		–	-.32 (<.001)**	-.37 (<.001)**	.43 (<.001)**	-.02 (.863)	-.20 (.033)*	.04 (.726)	.11 (.301)
**3**			–	.55 (<.001)**	-.66 (<.001)**	.00 (.981)	-.10 (.272)	-.46 (<.001)**	-.13 (.223)
**4**				–	-.65 (<.001)**	.05 (.563)	.25 (.007)*	.00 (.992)	-.30 (.003)*
**5**					–	.07 (.442)	-.20 (.033)*	.14 (.159)	.13 (.220)
**6**						–	.00 (.989)	.03 (.782)	.04 (.738)
**7**							–	.20 (.043)*	-.26 (.012)*
**8**								–	-.49 (<.001)*

* Significant at *p *< .05, uncorrected.

** Significant at *p *< .05 after Bonferroni's correction.

CLBP, prevalence of chronic low back pain; CNP, prevalence of chronic neck pain; GCI, Global Collectivism Index; HOF-PD, Hofstede Index of Power Distance; HOF-IC, Hofstede Index of Individualism-Collectivism; HOF-MF, Hofstede Index of Masculinity-Femininity; HOF-UAI, Hofstede Index of Uncertainty Avoidance; HOF-LTO, Hofstede Index of Long-Term Orientation; HOF-IVR, Hofstede Index of Indulgence vs. Restraint.

The prevalence of chronic neck pain was also negatively correlated with the GCI and positively correlated with the Hofstede index of individualism-collectivism. Though the strength of these associations was weaker than for low back pain, it remained significant after Bonferroni's correction. Neck pain was also negatively correlated with Power Distance and Uncertainty Avoidance, but only the former association was significant after correction. Overall, these results suggest that the prevalence both chronic low back pain and neck pain is lower in countries with a collectivistic orientation and a higher Power Distance; low back pain alone was also associated with higher Long-Term Orientation.

Bivariate correlations between measures of religiosity and the prevalence of both pain disorders are presented in Table 3. The prevalence of chronic low back pain was negatively correlated with all four measures of religiosity: this association was weak for affiliation, moderate for religious attendance and daily prayer, and strong for the importance assigned to religion. All these associations survived correction for multiple comparisons. The prevalence of chronic neck pain was also negatively correlated with all measures of religiosity; however, the strength of these associations was weaker, and only the associations with religious attendance and the importance assigned to religion were significant after applying Bonferroni's correction. These results suggest that countries with higher self-reported measures of religious belief and practice have lower levels of chronic low back pain and neck pain, particularly the former. It can also be noted from Table 3 that there was a strong degree of multicollinearity (*r *= .84 to.93) between the reported values for religious attendance, prayer and importance.

**Table 3:** Bivariate correlations between measures of religious belief and practice and the national prevalence of chronic low back and neck pain.

Variable	1 CLBP	2 CNP	3 REL-AFF	4 REL-ATT	5 REL-PRAY	6 REL-IMP
**1**	–	.55 (<.001)**	-.34 (<.001)**	-.64 (<.001)**	-.62 (<.001)**	-.70 (<.001)**
**2**		–	-.31 (.002)*	-.35 (<.001)**	-.30 (.002)*	-.34 (<.001)**
**3**			–	.53 (<.001)**	.62 (<.001)**	.67 (<.001)**
**4**				–	.85 (<.001)**	.90 (<.001)**
**5**					–	.93 (<.001)**

* Significant at *p *< .05, uncorrected.

** Significant at *p *< .05 after Bonferroni's correction.

CLBP, prevalence of chronic low back pain; CNP, prevalence of chronic neck pain; REL-AFF, percentage reporting religious affiliation; REL-ATT, percentage reporting weekly attendance at religious services; REL-PRAY, percentage reporting daily prayer; REL-IMP, percentage that consider religion important or very important.

Correlations between the aforementioned variables and the possible confounding or interacting variables included in this study are presented in Supplementary Material Table S2. In these analyses, the prevalence of chronic low back pain was positively correlated with the prevalence of anxiety, obesity, insufficient physical activity, tobacco use and alcohol use. A similar pattern was observed for chronic neck pain, though the associations with insufficient physical activity and alcohol use did not reach statistical significance. Unexpectedly, both types of chronic pain showed a negative correlation with the prevalence of depression.

Among cultural dimensions, the most significant associations with confounders were noted for the Global Collectivism Index (positive correlation with depression, negative correlation with all other variables) and with Hofstede's indices of Power Distance (negative correlation with anxiety disorders and alcohol use), Uncertainty Avoidance (positive correlation with obesity, insufficient physical activity, tobacco use and alcohol use), and Long-Term Orientation (negative correlation with anxiety and depression, positive correlation with tobacco and alcohol use). All four measures of religiosity were positively correlated with the prevalence of depression, while the three measures of religious attendance, prayer and importance assigned to religion were negatively correlated with the prevalence of obesity, tobacco use and alcohol use. These analyses suggest that the variables selected for these analyses do represent genuine confounders, being significantly correlated with both cultural and religious indices and with the prevalence of both types of pain.

Supplementary Material Table S3 summarizes the correlations between cultural dimensions and measures of religiosity. All measures of religiosity were positively correlated with the GCI, and these associations crossed the threshold for multicollinearity (*r *> .8) for daily prayer and for the importance accorded to religion. Among Hofstede's dimensions, all measures of religiosity were positively correlated with Power Distance, and negatively correlated with Individualism and Long-Term Orientation.

### Partial correlation analyses

For partial correlation analyses involving the prevalence of chronic low back pain, adjustments were made for all six confounding factors, as they were all significantly associated with this outcome. For those involving chronic neck pain, adjustments were made only for the four confounders—depression, anxiety, obesity and tobacco use—associated with this variable. The results of the partial correlation analyses are presented in Table 4.

**Table 4:** Partial correlation analyses of the national prevalence of chronic low back and neck pain and cultural and religious indices, adjusted for confounders.

Variable	GCI	HOF-PD	HOF-IC	HOF-MF	HOF-UAI	HOF-LTO	HOF-IVR	REL-AFF	REL-ATT	REL-PRAY	REL-IMP
**CLBP** †	-.27 (.002)*	-.02 (.851)	.29 (.006)*	.04 (.689)	-.02 (.884)	.24 (.040)*	.04 (.718)	-.05 (.633)	-.12 (.307)	-.16 (.153)	-.29 (.008)*
**CNP** ††	.01 (.876)	-.20 (.045)*	.27 (.008)*	-.01 (.918)	-.34 (<.001)*	-.03 (.790)	.13 (.256)	-.21 (.041)*	-.13 (.215)	-.13 (.217)	-.17 (.102)

^†^ Adjusted for depression, anxiety disorders, obesity, insufficient physical activity, tobacco use and alcohol use.

^††^ Adjusted for depression, anxiety disorders, obesity and tobacco use.

* Significant at *p *< .05.

CLBP, prevalence of chronic low back pain; CNP, prevalence of chronic neck pain; GCI, Global Collectivism Index; HOF-PD, Hofstede Index of Power Distance; HOF-IC, Hofstede Index of Individualism-Collectivism; HOF-MF, Hofstede Index of Masculinity-Femininity; HOF-UAI, Hofstede Index of Uncertainty Avoidance; HOF-LTO, Hofstede Index of Long-Term Orientation; HOF-IVR, Hofstede Index of Indulgence vs. Restraint; REL-AFF, percentage reporting religious affiliation; REL-ATT, percentage reporting weekly attendance at religious services; REL-PRAY, percentage reporting daily prayer; REL-IMP, percentage that consider religion important or very important.

In the first partial correlation analysis, after adjustment for confounders, the prevalence of chronic low back pain was significantly and negatively correlated with the Global Collectivism Index (partial *r *= -.27, *p *= .002) and the percentage of those who considered religion important (partial *r *= -.29, *p *= .008), and positively correlated with the Hofstede indices of Individualism-Collectivism (partial *r* = .28, *p *= .006) and Long-Term Orientation (partial *r *= .24, *p *= .040).

In the second partial correlation analysis, the prevalence of chronic neck pain was significantly negatively correlated with the Hofstede indices of Power Distance (partial *r *= -.20, *p *= .045), Uncertainty Avoidance (partial *r *= -.34, *p *< .001) and the percentage of those reporting a religious affiliation (partial *r *= -.21, *p *= .041) and positively correlated with the Hofstede index of Individualism-Collectivism (partial *r* = .27, *p *= .008).

### Multivariate analyses

Two multivariate linear regression analyses were carried out. When selecting variables for these analyses, two issues related to multicollinearity arose. First, there was significant multicollinearity between three of the four measures of religiosity. To address this, a composite index of religiosity was constructed by taking the arithmetic mean of these three variables, and this measure was used in the multivariate analyses if issues related to multicollinearity arose in this context. This composite index was significantly and negatively correlated with the prevalence of both low back pain (*r *= -.68, *p *< .001) and neck pain (*r *= -.35, *p *< .001). Second, there was significant multicollinearity between the GCI and two of the measures of religiosity. To circumvent this problem, the Hofstede index of Individualism-Collectivism was used instead of the GCI in the multivariate analyses. The complete results of both multivariate analyses are presented in Table 5.

**Table 5:** Multivariate linear regression analyses of cultural and religious variables associated with the prevalence of chronic low back and neck pain.

Dependent variable	Goodness of fit (F)	Degrees of freedom	Independent variables	Regression coefficient (*β*)	Significance level	Variance inflation factor	Percentage of variance explained (adjusted *R*^2^)
**CLBP**	13.38*	79	HOF-PD HOF-IC HOF-UAI HOF-LTO REL-AFF REL-COMP	.11 .49 .11 .12 .20 -.44	.356 < .001* .283 .309 .077 .006*	2.24 2.48 1.49 2.03 1.98 3.66	.485
**CNP**	6.81*	86	HOF-PD HOF-IC HOF-UAI REL-AFF REL-COMP	-.14 .26 -.25 .05 -.13	.272 .084 .029* .732 .404	1.93 2.53 1.46 1.96 2.62	.253

* Significant at *p *< .05.

CLBP, prevalence of chronic low back pain; CNP, prevalence of chronic neck pain; HOF-PD, Hofstede Index of Power Distance; HOF-IC, Hofstede Index of Individualism-Collectivism; HOF-UAI, Hofstede Index of Uncertainty Avoidance; HOF-LTO, Hofstede Index of Long-Term Orientation; REL-AFF, percentage reporting religious affiliation; REL-COMP, composite index of religiosity (attendance, prayer and importance).

In the first multivariate analysis, the estimated prevalence of chronic low back pain was the dependent variable, and the following independent variables were included in the model: Hofstede's indices of Power Distance, Individualism-Collectivism, Uncertainty Avoidance and Long-Term Orientation, Religious Affiliation, and the composite index of religiosity. The overall model was statistically significant, and explained around 48% of the variance in the prevalence of chronic low back pain (*R*^2^ = .524, adjusted *R*^2^ = .485). In this model, individualism was positively associated with the prevalence of low back pain (*β* = .49, *p *< .001), while the composite index of religiosity was negatively associated with this outcome (*β* = -.44, *p *= .006).

In the second multivariate analysis, the estimated prevalence of chronic neck pain was the dependent variable, and the following independent variables were selected for analysis: Hofstede's indices of Power Distance, Individualism-Collectivism and Uncertainty Avoidance, Religious Affiliation, and the composite index of religiosity. The overall model attained statistical significance, and explained around 25% of the variance in the prevalence of chronic neck pain (*R*^2^ = .296, adjusted *R*^2^ = .253). In this model, only one individual variable—Uncertainty Avoidance—was negatively associated with the prevalence of this type of pain (*β* = -.25, *p *= .029), though there was a trend towards a positive association with individualism (*β* = .26, *p *= .084). Variance inflation factors were below 4 for all variables in both models, indicating a low risk of multicollinearity.

In both the aforementioned models, confounding factors were not included, as this would have led to a relatively low number of subjects per independent variable. Nevertheless, additional linear regression analyses were carried out for exploratory purposes, including those confounding variables that were significantly (*p *< .05) associated with each outcome, as even with a relatively low subject-to-variable ratio, meaningful associations may be identified (72). These analyses are presented in Table 6. In the first of these analyses, the prevalence of chronic low back pain was the dependent variable, and the independent variables included the seven variables from the prior model, as well as the prevalence of depression, anxiety disorders, obesity, insufficient physical activity, tobacco use and alcohol use. In this analysis, significant concerns regarding multicollinearity (VIF = 6.64) were identified for the composite index of religiosity; therefore, this variable was excluded and the analysis repeated. This model showed a marginal increase in percentage of variance explained (adjusted R^2^ = .504) and was significant overall. The individual variables significantly associated with chronic low back pain were individualism (*β* = .40, *p *= .013), depression (*β* = -.23, *p *= .048), anxiety disorders (*β* = .26, *p *= .031) and alcohol use (*β* = .28, *p *= .018). In the second analysis, the prevalence of chronic neck pain was the dependent variable, and the independent variables included were the five from the previous model, as well as the prevalence of depression, anxiety disorders, obesity and tobacco use. As in the previous case, the composite index of religiosity had to be excluded due to a VIF of 4.28. This model showed a substantial increase in the percentage of variance explained (adjusted *R*^2^ = .402) and was significant overall. The individual variables associated with chronic neck pain were uncertainty avoidance (*β* = -.32, *p *= .004), anxiety disorders (*β* = .47, *p *< .001) and tobacco use (*β* = .22, *p *= .023).

**Table 6:** Multivariate linear regression analyses of cultural and religious variables associated with the prevalence of chronic low back and neck pain, including confounding factors.

Dependent variable	Goodness of fit (F)	Degrees of freedom	Independent variables	Regression coefficient (β)	Significance level	Variance inflation factor	Percentage of variance explained (adjusted *R*^2^)
**CLBP**	7.55*	71	HOF-PD HOF-IC HOF-UAI HOF-LTO REL-AFF DEP ANX OB IN-PA TOB ALC	.13 .40 .05 .19 .10 -.23 .26 .08 .07 .08 .28	.321 .013* .650 .159 .401 .048* .031* .518 .702 .464 .018*	2.56 3.60 2.01 2.56 1.79 1.83 1.98 2.25 1.42 1.69 1.93	.504
**CNP**	7.88*	82	HOF-PD HOF-IC HOF-UAI REL-AFF DEP ANX OB TOB	-.06 .12 -.32 -.03 -.05 .47 -.04 .22	.646 .417 .004* .761 .655 < .001* .762 .023*	2.12 2.81 1.54 1.51 1.45 1.58 1.86 1.22	.402

* Significant at *p *< .05.

CLBP, prevalence of chronic low back pain; CNP, prevalence of chronic neck pain; HOF-PD, Hofstede Index of Power Distance; HOF-IC, Hofstede Index of Individualism-Collectivism; HOF-UAI, Hofstede Index of Uncertainty Avoidance; HOF-LTO, Hofstede Index of Long-Term Orientation; REL-AFF, percentage reporting religious affiliation; REL-COMP, composite index of religiosity (attendance, prayer and importance); DEP, prevalence of depression; ANX, prevalence of anxiety disorders; OB, prevalence of obesity; IN-PA, prevalence of insufficient physical activity; TOB, prevalence of tobacco use; ALC, prevalence of alcohol use.

## Discussion

Chronic musculoskeletal pain is a paradigmatic example of a group of disorders requiring a biopsychosocial approach to treatment (73). Though most patients are offered biomedical treatments, both pharmacological and surgical, these are often ineffective or only partially effective, and some of the approaches that were often used in the past, such as opioid analgesics, are gradually being abandoned due to their unfavorable risk-to-benefit ratio (25). There is a substantial body of evidence linking psychological, social and religious/spiritual factors to various aspects of this group of conditions. The current study was conducted against this background, with the aim of identifying meaningful associations between cross-national variations in culture and religion and the prevalence of two common disorders—chronic low back pain and chronic neck pain—belonging to this group.

### Cultural dimensions and pain prevalence

In bivariate analyses, the cultural dimensions of individualism-collectivism and power distance were significantly associated with the prevalence of both types of pain: broadly speaking, these conditions were more common in countries whose culture was characterized by a lower power distance and higher individualism. These findings are consistent with those of an earlier meta-analysis by Kroska (52), who found that these two dimensions of culture significantly mediated the association between respondents' fear-related avoidance and the severity of pain reported by them. Power distance is a measure of the extent to which institutionalized inequality and hierarchy is accepted as normal in a given society. In such societies, individuals may be more tolerant of acts and situations that could be perceived as unjust in others. Individuals' perceptions of injustice have been identified as an important predictor of symptom severity, depression, anxiety and disability in patients with musculoskeletal pain, independent of age and pain duration (74, 75). It is therefore plausible that cultural power distance may influence chronic pain through the intermediate variable of perceived injustice. However, though the negative relationship between power distance and pain was significant for chronic neck pain even after adjusting for confounders, it was not significant in the multivariate analysis; therefore, hypotheses such as the one outlined above should be considered speculative.

The two measures of individualism-collectivism showed somewhat different associations with the prevalence of chronic low back and neck pain. In the case of the Global Collectivism Index, this association was significant after adjusting for confounders only in the case of low back pain, whereas Hofstede's index of individualism-collectivism survived these adjustments for both types of pain. In multivariate analyses, the association between the Hofstede index and the prevalence of pain was significant only for chronic low back pain. Thus, while individualism-collectivism may account for some of the cross-national variation in the prevalence of these pain disorders, the consistency and strength of this association varies depending on how this cultural dimension is measured. Several factors may account for the inverse association between collectivism and chronic low back and neck pain. Collectivistic cultures are generally characterized by higher levels of collective coping (43, 76) and family and community support (77, 78), which may be associated with increased psychological well-being (76, 79). Social support has been identified as an important predictor of outcome in patients with chronic musculoskeletal pain, including low back and neck pain, affecting both the course and severity of pain and the likelihood of returning to work (80–82). Collectivistic cultural values may also be positively associated with key psychological processes such as self-regulation (83), which can influence both the perception of musculoskeletal pain and the disability associated with it (84, 85). These psychological and social factors may explain why collectivism appears to have a protective effect against chronic low back and neck pain: however, the current study was not designed to examine the mediating effects of such variables.

Among the other cultural dimensions studied, long-term orientation was positively correlated with the prevalence of chronic low back pain in both the direct and partial correlation analyses. However, this association was not significant in multivariate models, and may have been due to the negative correlation between long-term orientation and collectivism (*r_(GCI, LTO)_ = *-.46, *p *< .001). Uncertainty avoidance was negatively correlated with the prevalence of chronic neck pain after adjusting for confounders, and this finding was replicated in both multivariate models. A prior analysis of uncertainty avoidance at a cross-national level found that this dimension of culture was positively associated with experiences of “pain, worry and sadness”; however, this study only involved thirty high-income countries with predominantly individualistic cultures (86). In contrast, a study examining the association between Hofstede's cultural dimensions and quality of life found no significant correlation between pain-related quality of life and uncertainty avoidance (87). Uncertainty avoidance measures the extent to which a society is able to tolerate ambiguous or uncertain situations; high scores on this dimension imply that a country's culture would have rigid codes of conduct and be intolerant of unconventional ideas or behaviour (88). In a study of patients with chronic musculoskeletal pain, a measure of the complexity of each patient's psychosocial situation was found to be associated with altered methylation of the brain-derived neurotrophic factor (BDNF) gene (89). BDNF is an important regulator of neural plasticity, and plays a central role in several psychological processes related to chronic pain, including stress response, learning and memory. It has also been associated with chronic musculoskeletal pain in particular, perhaps through alterations in central pain processing (90, 91). It is possible that individuals living in cultures with well-defined norms and rules (in other words, high uncertainty avoidance) may experience less complex psychosocial circumstances, and that this might be a protective factor against chronic neck pain: however, such an explanation must be considered speculative.

### Measures of religious belief and practice and pain prevalence

In this study, all four measures of religious belief and practice—affiliation, attendance at religious services, prayer, and the importance assigned to religion—were negatively correlated with the prevalence of chronic low back pain and neck pain, though stronger correlations were observed for low back pain. However, after adjustment for possible confounders, only two associations remained significant: chronic low back pain was negatively correlated with the importance given to religion in one's life, and chronic neck pain was negatively correlated with religious affiliation. These findings could not be replicated in the multivariate analyses. These results are consistent with the mixed findings of the available literature on the links between religion / spirituality and these types of pain (55–61). It is likely that the weak and inconsistent results related to religion obtained in this study reflect methodological limitations. First, there was significant multicollinearity between three of the four measures of religiosity reported in the Pew Research Center's publication; second, there was significant multicollinearity between these measures of religiosity and the Global Collectivism Index. This led to a loss of precision and specificity in the partial correlation and multivariate analyses. Secondly, the outcome variables in this study were the prevalence of each type of chronic pain, whereas prior research has found religious variables to influence the course, rather than the occurrence, of conditions such as chronic low back and neck pain. Third, there are several aspects of religiosity and spirituality—such as positive and negative religious coping, spiritual experiences, forgiveness and support from a religious group or community—that could be relevant to the onset, severity and chronicity of low back or neck pain, and which were not captured by questions asked in the Pew Research Center surveys. In this context, the results obtained by Rippentrop et al. in a sample of patients with chronic musculoskeletal pain are of particular interest. The findings of this study suggest that specific aspects of religious or spiritual belief and practice could have both negative and positive influences on the health of patients with this type of pain—as stated by the authors, “religion/spirituality may have both costs and benefits” in this context (92). Subsequent research has confirmed the “double-sided” nature of the relationship between religiosity and chronic pain—for example, an association between prayer and greater impairment, interference, and depression associated with chronic pain has been reported in Swedish adults (93), while a study of older adults from the United States found a longitudinal association between religious service attendance and decreased pain severity over a period of three years (94). In the absence of further longitudinal data, it is not possible to draw direct causal inferences from such results: for example, do people experience more pain-related distress when they pray more frequently, or are they more likely to turn to prayer when they experience intractable or disabling pain which does not respond to standard medical treatment? (95). It is certainly possible that certain aspects of religiosity may be protective against chronic low back and neck pain, but this cannot be confirmed in the current study. The variability in the results obtained to date highlight the need for better measures of the different facets of religion and spirituality, their relationship to the persistence of musculoskeletal pain and the disability associated with it, and the possible mediating role of psychological variables such as affective states and pain-related beliefs (96).

### Relationship between other variables and pain prevalence

In both multivariate models, certain variables remained associated with the prevalence of chronic pain independent of cultural and religious variables. For chronic low back pain, these were the prevalence depression, anxiety disorders and alcohol use; for chronic neck pain, these were the prevalence of anxiety disorders and tobacco use. The positive association between anxiety disorders and both pain types is consistent with the existing literature: anxiety is associated with greater musculoskeletal pain severity (97) and negative pain-related cognitions (98), and there is a high degree of comorbidity between chronic musculoskeletal pain and anxiety disorders (99). In this study, the prevalence of anxiety disorders was also positively correlated with individualism and negatively correlated with power distance, suggesting that these cultural factors are associated with variations in the prevalence of both pain and anxiety. Unexpectedly, the prevalence of depression was negatively associated with the prevalence of chronic low back pain, even in the multivariate analysis. While this finding appears to contradict the existing literature, it should be noted that some studies have found that depression failed to predict variations in either the prevalence or the severity of chronic low back pain (100, 101). Moreover, the contradiction may be more apparent than real. In some non-Western cultures characterized by different idioms of distress, and in which a certain stigma is attached to mental disorders depression may present to the physician as chronic or intractable musculoskeletal pain (102). Such a presentation is referred to as “masked depression” and could account for the apparent inverse relationship between the prevalence of depression and chronic low back pain in these countries (103). In support of this contention, the negative correlation between chronic low back pain and depression was no longer significant when adjusting for power distance and collectivism (*r *= -.11, *p *= .253). Given the evidence of a genetic link between depression and both these types of pain (14), it is possible that cultural factors may interact with an innate genetic vulnerability, leading to phenotypic variations in which some patients present predominantly with depression and others with chronic back or neck pain. This result highlights the need for a more culturally sensitive assessment of depression in patients presenting with a primary complaint of chronic pain, particularly in low- and middle-income countries.

### The possible confounding effect of healthcare systems and records

The use of prevalence estimates as measures of chronic low back and neck pain in the current study raises a further issue. Prevalence estimates in the Global Burden of Disease Study are based on a wide range of sources. These include published literature on the prevalence of each disorder, as well as data obtained from clinical trials, government records, hospital records and epidemiological surveillance (104). Cultural and religious factors have been shown to influence key components of individuals' interactions with healthcare systems, and may thereby influence the quality of the data obtained from these sources. High Power Distance is associated with a lower level of trust and satisfaction in healthcare systems (105); this may lead to reduced help-seeking and an underestimation of prevalence. Individualism-Collectivism, Uncertainty Avoidance and Long-Term Orientation can influence the quality of care received in primary settings (106), which could also influence patients' willingness to seek help for chronic pain. Likewise religious and spiritual beliefs could lead patients to seek complementary and alternative methods of care for their chronic low back or neck pain (61, 107); this could lead to underestimates of prevalence if these are based on hospital data. For these reasons, it is possible that the current study may have captured cross-cultural variations in help-seeking or data quality for chronic low back or neck pain, rather than in the actual prevalence of these conditions.

### Differences between factors associated with chronic low back pain and neck pain

Another facet of this study's findings that merits discussion is the divergence in the cultural correlates of chronic low back pain and neck pain. Despite the evidence of a possible epidemiological and genetic overlap between these conditions, the majority of patients presenting with one of these types of pain do not experience the other, and they are rightly considered distinct types of chronic musculoskeletal pain. The risk factors for each of these conditions also differ in certain key aspects. For example, chronic neck pain is common in office workers and related to specific postural and ergonomic factors (108, 109), while chronic low back pain is common both in sedentary workers and those engaged in physical labor, and may be associated with factors such as physical exertion and toxin exposure in the latter group (110, 111). Besides influencing individuals' opportunities for employment and their working environment, cultural factors are correlated with economic factors such as national income and industrialization, at a national level (67, 112). It is possible that distinct cultural factors may influence chronic low back or neck pain through their associations with a country's economy and working conditions, though this could not be directly examined in the current study.

### Strengths and limitations of the current study

This study is the first to examine cross-national variations in the prevalence of chronic low back and neck pain in relation to variations in cultural values and religious affiliation and practice. Data on each variable of interest was obtained from research studies of databases of good quality, which provided data on a large number of countries across income groups. In addition, efforts were made to minimize the risk of spurious associations by adjusting for key confounding factors identified in the existing literature.

Nevertheless, the current study is subject to certain important limitations. First, due to its cross-sectional design, it cannot draw any firm conclusions regarding causality; only associations between cultural dimensions and chronic back and neck pain can be inferred. Second, as countries were the unit of analysis, these findings cannot be directly generalized or applied to individuals. Third, it was not possible to capture variations within a country (such as urban-rural differences, or cultural variations in a multi-ethnic or multi-religious society) from the available data. Fourth, the data sources used for the GBD, though comprehensive, are not completely free of bias, as discussed in the preceding section. Fifth, though an attempt was made to correct for confounders, there are several other key confounding variables identified in the literature, such as social support, stress and workplace culture, that could not be assessed due to a lack of cross-national data. Finally, the variables used to measure various aspects of religion were of limited value in the analyses, due to a high degree of multicollinearity both among them, and between them and measures of collectivism.

### Practical implications of the current results

Results obtained from analyses at the level of countries cannot be applied directly to individuals. Nevertheless, the results of this study have significance from a clinical perspective, as they highlight the need to consider variations in cultural and religious values and practices when managing patients with chronic pain. As mentioned above, the successful management chronic musculoskeletal pain requires a holistic approach that goes beyond the prescription of specific medical treatments. Culture and religion can shape the experience of chronic pain, the meaning attached to it, the affective responses associated with it (such as anxiety, depression, and anger) and the willingness to seek and adhere to specific types of treatment. The responses of both caregivers and the patient's immediate community to their suffering are also shaped by cultural and religious beliefs. This becomes especially relevant in contemporary medical practice, where migration and globalization often necessitate a greater degree of cultural sensitivity and competence on the part of healthcare professionals managing a patient with a chronic disorder. Awareness of the way in which variations in national cultural values, or in religious beliefs, influence these facets of chronic pain can foster the development of a better therapeutic relationship, enhance concordance between clinicians and patients, and possibly reduce the inappropriate use of treatments with a low risk-benefit ratio. Knowledge of cross-national variations in values and beliefs can also aid the judicious selection of specific treatment approaches, such as spiritually-informed cognitive or behavioral interventions.

## Conclusions

Despite certain limitations, the current study has identified a possible influence of cultural values on cross-national variations in two common and disabling forms of chronic musculoskeletal pain. Though this study's results should be considered provisional, they are consistent with the growing body of literature highlighting the importance of cultural and religious factors in the pathogenesis and treatment of chronic low back and neck pain. Cross-national research in individual subjects would help to further elucidate the relative importance of these factors in a given case, as well as the biological and psychological processes through which they may exert beneficial or harmful results. The current study also highlights the need for further examination of the links between religion / spirituality and chronic musculoskeletal pain across different countries and regions, with a focus on the subjective aspects of religiosity instead of measures of affiliation or attendance which may reflect cultural norms rather than religious or spiritual conviction.

## Data Availability

The original contributions presented in the study are included in the article/**Supplementary Material**, further inquiries can be directed to the corresponding author.
