# Supplementary material for: The influence of cultural and religious factors on cross-national variations in the prevalence of chronic back and neck pain: an analysis of data from the global burden of disease 2019 study
Source: Front Pain Res (Lausanne). 2023 May 25;4:1189432. doi: 10.3389/fpain.2023.1189432 (PMC10248050; doi:10.3389/fpain.2023.1189432)
Supplement: Supplementary file 1 [file Datasheet1.pdf]

Supplementary Table 1: Descriptive statistics for all study variables

Variable	Mean (Standard Deviation)	Maximum	Minimum
Chronic low back pain, prevalence (%)	8.11 (1.61)	13.74	5.37
Chronic neck pain, prevalence (%)	2.28 (1.14)	5.55	0.96
Global Collectivism Index	0.11 (0.79)	1.92	-1.85
Hofstede Index of Power Distance	66.38 (20.65)	100	11
Hofstede Index of Individualism-Collectivism	37.73 (21.18)	91	6
Hofstede Index of Masculinity-Femininity	46.66 (17.69)	100	5
Hofstede Index of Uncertainty Avoidance	66.71 (21.20)	100	8
Hofstede Index of Long-Term Orientation	44.28 (24.08)	100	0
Hofstede Index of Indulgence Versus Restraint	45.44 (23.40)	100	0
Religious affiliation (%)	88.06 (17.22)	100	13
Religious attendance, weekly (%)	38.68 (27.05)	89	1
Daily prayer (%)	49.22 (27.46)	96	1
Religion considered important (%)	54.41 (31.30)	98	3
Depression, estimated prevalence (%)	2.74 (0.92)	5.55	0.82
Anxiety disorders, estimated prevalence (%)	4.39 (1.20)	8.79	2.14
Obesity, prevalence (%)	19.96 (11.41)	61	2.1
Tobacco use, prevalence (%)	20.71 (9.88)	49.2	3.6
Alcohol use, prevalence (%)	5.82 (4.05)	14.26	0
Insufficient physical activity, prevalence (%)	28.12 (10.95)	67	5.5

Supplementary Table 2: Bivariate correlations between the prevalence of chronic low back and neck pain, cultural and religious variables, and possible confounding factors

Variable	Depression, prevalence	Anxiety disorders, prevalence	Obesity, prevalence	Insufficient physical activity, prevalence	Tobacco use, prevalence	Alcohol use, prevalence
**Chronic low back pain, prevalence**	-.28 (<.001)**	.36 (<.001)**	.40 (<.001)**	.23 (.003)*	.49 (<.001)**	.49 (<.001)**
**Chronic neck pain, prevalence**	-.22 (<.001)*	.47 (<.001)**	.29 (<.001)**	.14 (.086)	.40 (<.001)**	.10 (.184)
**Global Collectivism Index**	.21 (.005)*	-.40 (<.001)**	-.44 (<.001)**	-.35 (<.001)**	-.27 (<.001)*	-.67 (<.001)**
**Hofstede Index of Power Distance**	-.12 (.197)	-.42 (<.001)**	-.15 (.102)	-.09 (.380)	.03 (.731)	-.41 (<.001)**
**Hofstede Index of Individualism-Collectivism**	.18 (.058)	.42 (<.001)**	.34 (<.001)**	.19 (.051)	.19 (.058)	.47 (<.001)**
**Hofstede Index of Masculinity-Femininity**	-.16 (.094)	-.02 (.882)	.08 (.419)	.23 (.019)*	-.01 (.941)	-.01 (.889)
**Hofstede Index of Uncertainty Avoidance**	-.06 (.492)	.06 (.555)	.35 (<.001)**	.22 (.027)*	.23 (.021)*	.19 (.044)*
**Hofstede Index of Long-Term Orientation**	-.42 (<.001)**	-.23 (.020)*	-.17 (.097)	-.10 (.341)	.55 (<.001)**	.43 (<.001)**
**Hofstede Index of Indulgence Versus Restraint**	.19 (.062)	.30 (.003)*	.05 (.618)	.02 (.838)	-.50 (<.001)**	.02 (.822)
**Religious affiliation (%)**	.25 (.009)*	-.14 (.156)	-.05 (.624)	-.05 (.609)	-.17 (.103)	-.45 (<.001)**
**Religious attendance, weekly (%)**	.35 (<.001)**	-.26 (.009)*	-.53 (<.001)**	-.21 (.052)	-.61 (<.001)**	-.58 (<.001)**
**Daily prayer (%)**	.28 (.004)**	-.14 (.152)	-.29 (.003)*	.01 (.922)	-.53 (<.001)**	-.72 (<.001)**
**Religion considered important (%)**	.34 (<.001)**	-.17 (.093)	-.36 (<.001)**	-.11 (.316)	-.52 (<.001)**	-.67 (<.001)**

* Significant at $p < .05$, uncorrected.

** Significant at $p < .05$ with Bonferroni's correction.

Supplementary Table 3: Bivariate correlations between cultural dimensions and measures of religiosity

Variable	Global Collectivism Index	Hofstede –Power Distance	Hofstede – Individualism-Collectivism	Hofstede – Masculinity-Femininity	Hofstede – Uncertainty Avoidance	Hofstede – Long-Term Orientation	Hofstede – Indulgence versus Restraint
**Religious affiliation**	.60 (<.001)**	.38 (<.001)**	-.43 (<.001)**	.02 (.826)	.23 (.031)*	-.40 (<.001)**	-.12 (.295)
**Weekly attendance at religious services**	.79 (<.001)**	.32 (.004)*	-.48 (<.001)**	.10 (.385)	-.18 (.103)	-.59 (<.001)**	.19 (.096)
**Daily prayer**	.80 (<.001)**	.44 (<.001)**	-.57 (<.001)**	.02 (.885)	.00 (.974)	-.62 (<.001)**	.08 (.473)
**Consider religion important**	.84 (<.001)**	.41 (<.001)**	-.58 (<.001)**	.04 (.689)	-.08 (.445)	-.66 (<.001)**	.11 (.353)

* Significant at $p < .05$, uncorrected.

** Significant at $p < .05$ with Bonferroni's correction.
